# Comparative genome analysis of *Streptococcus infantarius* subsp. *infantarius* CJ18, an African fermented camel milk isolate with adaptations to dairy environment

**DOI:** 10.1186/1471-2164-14-200

**Published:** 2013-03-22

**Authors:** Christoph Jans, Rainer Follador, Mira Hochstrasser, Christophe Lacroix, Leo Meile, Marc J A Stevens

**Affiliations:** 1 Laboratory of Food Biotechnology, Institute of Food, Nutrition and Health, Schmelzbergstrasse 7, ETH Zurich, Zurich, CH, 8092, Switzerland

**Keywords:** *Streptococcus infantarius*, *Streptococcus bovis/Streptococcus equinus* complex, *Streptococcus thermophilus*, *Streptococcus gallolyticus* subsp*. macedonicus*, Dairy fermentation, Lactose metabolism, Africa, Camel, Health risk, *Streptococcus* virulence factors

## Abstract

**Background:**

*Streptococcus infantarius* subsp. *infantarius* (*Sii*) belongs to the *Streptococcus bovis/Streptococcus equinus* complex associated with several human and animal infections. *Sii* is a predominant bacterium in spontaneously fermented milk products in Africa. The genome sequence of *Sii* strain CJ18 was compared with that of other *Streptococcus* species to identify dairy adaptations including genome decay such as in *Streptococcus thermophilus*, traits for its competitiveness in spontaneous milk fermentation and to assess potential health risks for consumers.

**Results:**

The genome of *Sii* CJ18 harbors several unique regions in comparison to *Sii* ATCC BAA-102^T^, among others an enlarged exo- and capsular polysaccharide operon; *Streptococcus thermophilus-*associated genes; a region containing metabolic and hypothetical genes mostly unique to CJ18 and the dairy isolate *Streptococcus gallolyticus* subsp. *macedonicus*; and a second oligopeptide transport operon. Dairy adaptations in CJ18 are reflected by a high percentage of pseudogenes (4.9%) representing genome decay which includes the inactivation of the lactose phosphotransferase system (*lacIIABC*) by multiple transposases integration. The presence of *lacS* and *lacZ* genes is the major dairy adaptation affecting lactose metabolism pathways also due to the disruption of *lacIIABC*.

We constructed mutant strains of *lacS, lacZ* and *lacIIABC* and analyzed the resulting strains of CJ18 to confirm the redirection of lactose metabolism via LacS and LacZ.

Natural competence genes are conserved in both *Sii* strains, but CJ18 contains a lower number of CRISPR spacers which indicates a reduced defense capability against alien DNA. No classical streptococcal virulence factors were detected in both *Sii* strains apart from those involved in adhesion which should be considered niche factors. *Sii*-specific virulence factors are not described. Several *Sii*-specific regions encoding uncharacterized proteins provide new leads for virulence analyses and investigation of the unclear association of dairy and clinical *Sii* with human diseases.

**Conclusions:**

The genome of the African dairy isolate *Sii* CJ18 clearly differs from the human isolate ATCC BAA-102^T^. CJ18 possesses a high natural competence predisposition likely explaining the enlarged genome. Metabolic adaptations to the dairy environment are evident and especially lactose uptake corresponds to *S. thermophilus.* Genome decay is not as advanced as in *S. thermophilus* (10-19%) possibly due to a shorter history in dairy fermentations.

## Background

The putative pathogen *Streptococcus infantarius* subsp. *infantarius* (*Sii*) is a lactic acid bacterium (LAB) commonly associated with the gastrointestinal tract of animals and humans [1]. Additionally, *Sii* has been isolated from dairy products, feces (including the type strain ATCC BAA-102^T^ and isogenetic strain CCUG 43820^T^), human blood (n = 3) and human endocarditis (n = 3) [2-5]. Recently, it was identified as the predominant species in several spontaneously fermented African dairy products such as *suusac, gariss* and *fènè*[2,5-7] and in the Mexican fermented maize beverage *pozol*[8]. *Sii* belongs to the Lancefield group D *Streptococcus bovis/Streptococcus equinus* complex (SBSEC) which comprises the species *S. bovis, S. equinus, Streptococcus lutetiensis* (known as *Streptococcus infantarius* subsp*. coli*), *Streptococcus gallolyticus* subsp. *gallolyticus* (formerly *S. bovis* biotype I), *Streptococcus gallolyticus* subsp. *macedonicus, Streptococcus gallolyticus* subsp. *pasteurianus* and *Streptococcus alactolyticus*[3,4,9].

The SBSEC is commonly associated with many infectious diseases such as bacteremia, endocarditis and bloat [1]. Moreover, some members of the group, especially *S. gallolyticus* subsp*. gallolyticus*, are suspected to play a role in colonic cancer development [10,11], partly associated to increasing mRNA levels of IL-1, IL-8 and COX-2 in colorectal tissue, which contribute to inflammation caused tumor development [12]. Because of the high risk association of mainly *S. gallolyticus* subsp. *gallolyticus* with infectious diseases and cancer, research on virulence within the SBSEC group has largely focused on this species [12-16]. Virulence factors such as fibrinogen binding factor FimB, glucosyltransferase Gtf and pilus subunit B PilB have been identified in several SBSEC members [16-19]. Additionally, potential virulence factors such as adhesion proteins have been shown e.g. the surface protein histone-like protein A (HlpA), the “adhesion to collagen of the *S. bovis* group” (Acb) and “*S. bovis* group surface protein” (Sbs) [14,20]. However, many of these factors seem to be necessary for survival of SBSEC in the gastrointestinal tract and should therefore be considered as niche factors [21].

The pathogenicity of *Sii* is less elucidated. Potential pro-inflammatory proteins were detected in *Sii* and the species is also associated with non-colonic cancer [22,23]. In parallel to *S. gallolyticus* subsp. *gallolyticus*, a *Sii* strain isolated from feces of an infected baby was able to translocate across a polarized epithelial monolayer of Caco-2 cells, a property which potentially facilitates infection [24]. This ability was so far only demonstrated for a single *Sii* strain of clinical and not of food origin. In a recent and broad clinical study on 58 *S. bovis* strains, only the subspecies *S. infantarius* subsp. *coli* (n = 17), but not *Sii*, was isolated from blood of infected patients among 29 *S. gallolyticus* subsp. *gallolyticus* and 12 *S. gallolyticus* subsp. *pasteurianus*[10]. This suggests only a minor role of *Sii* in infectious diseases. Nevertheless, the predominance of *Sii* in African food fermentations [2-5] and, as a consequence, the ingestion of high amounts of viable cells of this species by the consumer demands further research to elucidate any potential pathogenic traits of this SBSEC member and possibly diverge dairy from clinical isolates.

*Streptococcus thermophilus* is the only streptococcal species recommended by the qualified presumption of safety (QPS) for use in fermented food products [25]. It displays an adaptation to the milk environment that is characterized by genome reduction, gene decay and loss of function, which is reflected by the high abundance of pseudogenes in all sequenced *S. thermophilus* genomes [26,27]. Genome reduction through loss or inactivation of virulence factors and long history of use contributed to the recognition of *S. thermophilus* by QPS, despite its close genetic relationship to the SBSEC [25-29]. Interestingly, *Streptococcus macedonicus* ACA-DC 198 (designated *S. gallolyticus* subsp. *macedonicus* in this study according to [3]), a Greek cheese isolate, displayed comparable genome decay to *S. thermophilus* and could indicate parallel evolutionary adaptation to the dairy environment in other members of the SBSEC and important contributions of certain members of the SBSEC to dairy fermentations in Europe [30].

The predominance and probably exclusive habitat of the African *Sii* variants in dairy fermentations suggests adaptation to the dairy environment similar to *S. thermophilus*[2,7]. This predominance seems directly related to the presence of a *gal-lac* operon in the African variant of *Sii*[7], a feature that is absent in other members of the SBSEC. Furthermore, African strains display a lactose fermentation pattern paralleling that of *S. thermophilus*[7]. The high prevalence of bacteriocin producers among African *Sii* isolates likely contributes to the predominance of *Sii* in African dairy fermentations [2].

In this work, we present the complete genome sequence of *Sii* CJ18 isolated as representative predominant strain from spontaneously fermented camel milk *suusac* from Kenya at over 10^8^ CFU mL^-1^. CJ18 does not produce bacteriocin-like inhibitory substances [2]. It was selected for genome sequencing due to genetic and metabolic evidence of a lactose fermentation pattern similar to *S. thermophilus* after studying of 3 different African *Sii* isolates [7]. A genomic comparison of strain CJ18 to other pathogenic and non-pathogenic streptococci was performed in order to identify dairy adaptations and potential virulence factors in CJ18. Our study provides new insight into streptococcal evolution in the previously untouched ecosystem of dairy fermentations in Africa and provides new insight on safety and occurrence of horizontal gene transfer (HGT) of streptococci in food fermentations.

## Results

### General genome properties

The genome of *Sii* CJ18 consists of a 1,988,420-bp circular molecule encoding 2050 genes of which 1867 encode for proteins [GenBank:CP003295, GenBank:CP003296] (Table 1) [31]. Comparison of genes with their homologues in other streptococcal genomes, resulted in detection of 97 (4.9%) genes that carry a deletion, insertion or premature stop, and that were therefore assigned as pseudogenes. Additionally, 19,829 bp of plasmid related DNA, designated pSICJ18-1, providing 35 coding DNA sequences (CDS) with only limited similarity to SBSEC sequences were detected. The nucleotide sequence (96-100% identity) and G + C mol%-content of 30 out of 35 CDS suggest a lactococcal origin [Additional file 1].

**Table 1:** **General features of the *Sii *CJ18 genome and other sequenced genomes of streptococci**

	***S. infantarius *subsp. *infantarius***		***S. gallolyticus *subsp. *gallolyticus***			***S. gallolyticus *subsp*. macedonicus***	***S. gallolyticus *subsp*. pasteurianus *ATCC 43144**	***S. agalactiae *2603 V/R**	***S. pyogenes *M1 GAS**	***S. pneumoniae *D39**	***S. thermophilus***		
	**CJ18**	**ATCC BAA-102**^**T**^	**ATCC 43143**	**ATCC BAA-2069**	**UCN34**	**ACA-DC 198**					**LMD-9**	**CNRZ 1066**	**LMG 18311**
length (bp)	1,988,420 + 19,829 pSICJ18-1	1,938,634	2,362,241	2,356,444	2,350,911	2,130,034 + 12,728 pSMA198	2,100,077	2,160,267	1,852,441	2,046,115	1,856,368	1,796,226	1,796,846
G + C (mol%)	37.6	37.6	37.5	37.6	37.6	37.6	37.4	35.6	38.5	39.7	39.1	39.1	39.1
genes	2050 + 35 pSICJ18-1	1988	2371	2410 + 21 pSGG1	2349	2280 + 17 pSMA198	2102	2276	1810	2069	2002 + 4 Plsm1 + 2 Plsm 2	2000	1973
pseudogenes/truncated proteins (%)^b)^	97 (4.9%)	n/a ^a)^	49 (2.1%)	0	37 (1.6%)	215 (9.8%)	157 (7.9%)	0	35 (2.0%)	82 (4.1%)	206 (10.8%)	~19%	~19%
protein (non tRNA/rRNA)	1867		2246	2309	2223	1977	1869	2124	1696	1914	1709	1915	1888
tRNA genes	68	46^c)^	60	80	71	70	61	80	60	58	67	67	67
rRNA genes	18	8^c)^	15	21	18	17	15	21	18	12	19	18	18
source	fermented camel milk *suusac*[31]	baby feces (HMP)	human clinical specimen, blood [32]	human clinical specimen, blood [33]	human clinical specimen, blood [13]	Greek Kasseri cheese [30]	human clinical specimen, blood [32]	human clinical specimen [34]	human clinical specimen [35]	human clinical specimen [36]	yogurt [27]	yogurt [26]	yogurt [26]

^a)^ n/a: Not available; ^b)^ Calculated according to: #pseudogenes/(#pseudogenes + total proteins)*100; ^c)^ The genome of ATCC BAA-102^T^ was only aligned to that of CJ18 but not completely assembled. tRNA and rRNA genes are possibly underestimated in the type strain as genome gaps were not closed. HMP: Human Microbiome Project http://www.hmpdacc.org.

**Figure 1:**
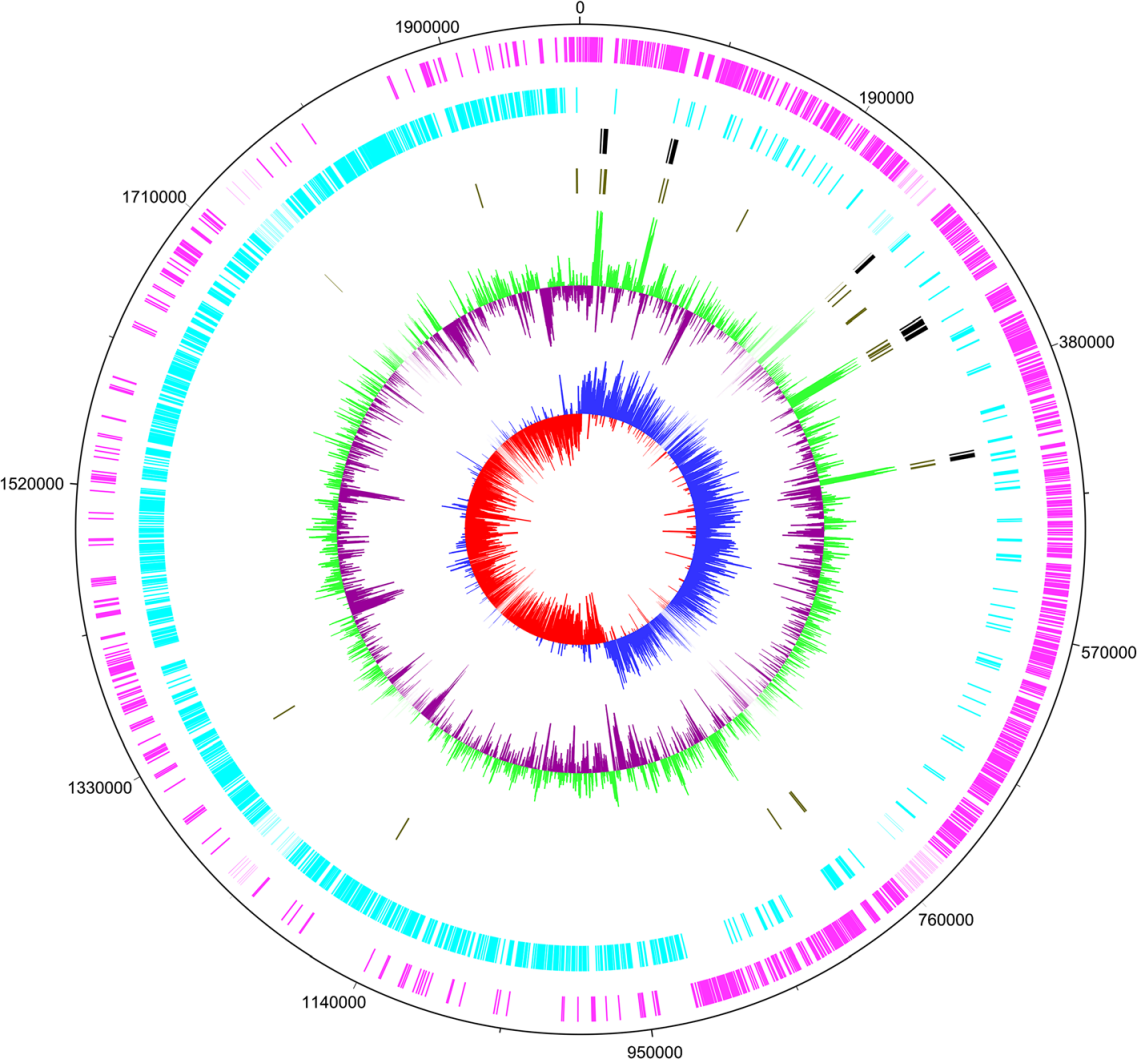
**Circular genome of *Sii *CJ18.** The inner most circle shows the GC-skew of higher (blue) and lower (red) than average followed by the GC-content with higher (green) and lower (dark purple) than average. The third (olive green) and fourth circle (black) display tRNA and rRNA, respectively. The two outermost circles indicate the position of coding sequences on the forward (pink) or reverse (ice blue) strand.

The origin of the genome of CJ18 was determined upstream of the *dnaA* gene and corresponds to the switch in GC-skew (Figure 1). However, a shift towards the 5 o’clock position was detected for the terminus position, as is reflected by a switch in the GC-skew and in the CDS-density on the forward and reverse strand (Figure 1), a feature also observed in *S. gallolyticus* subsp. *gallolyticus* ATCC 43143 and *S. gallolyticus* subsp. *pasteurianus* ATCC 43144 [32].

**Figure 2:**
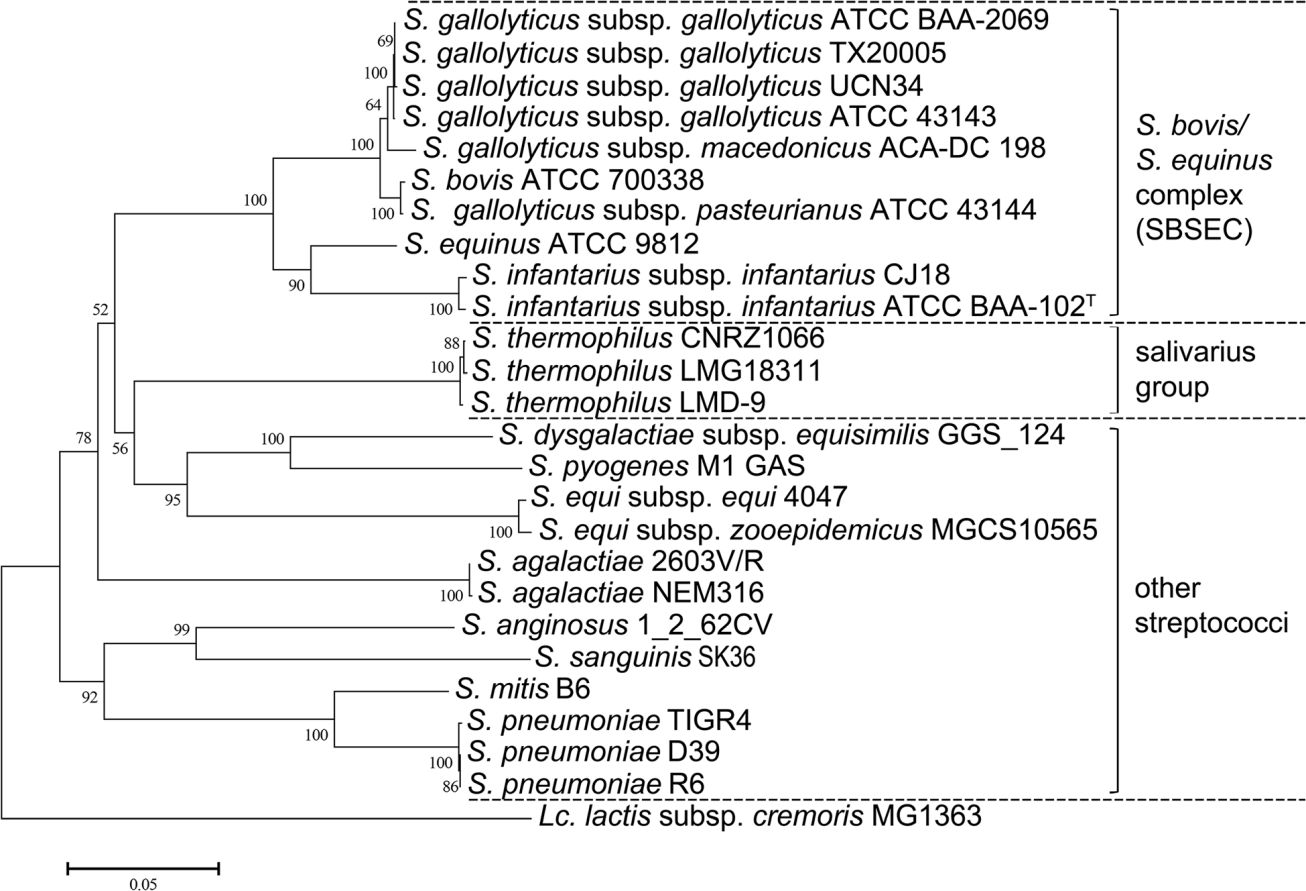
**Rooted phylogenetic tree calculated for *groEL *sequences of *Sii *CJ18 and related streptococci.** Rooted phylogenetic tree was calculated for the *groEL* genes of *Sii* CJ18, related SBSEC members and other streptococci. CJ18 was clearly positioned on the same branch as *Sii* ATCC BAA-102^T^ within the SBSEC. The same phylogenetic position of CJ18 was obtained for the 16S rRNA gene and *gyrB, recA, recN, rpoB, secA, secY* and *sodA* with *groEL*, *secY* and *recN* yielding highest bootstrap percentages (data not shown). The evolutionary distances indicated by the horizontal bar below the figure are in the units of the number of base substitutions per site.

The complete genome sequence was used to confirm the taxonomy of CJ18 through alignment and subsequent phylogenetic analysis using 16S rRNA and eight typical streptococcal genes (*groEL, gyrB, recA, recN, rpoB, secA, secY* and *sodA*). All genes clearly positioned CJ18 within the SBSEC on the same branch as its closest relative *Sii* ATCC-BAA-102^T^ (Figure 2, tree only shown for *groEL*). The highest bootstrap percentages were obtained for trees based on *groEL*, *recN* and *secY* sequences (data not shown).

### Comparison of CJ18 to ATCC BAA-102^T^ and other SBSEC strains

**Figure 3:**
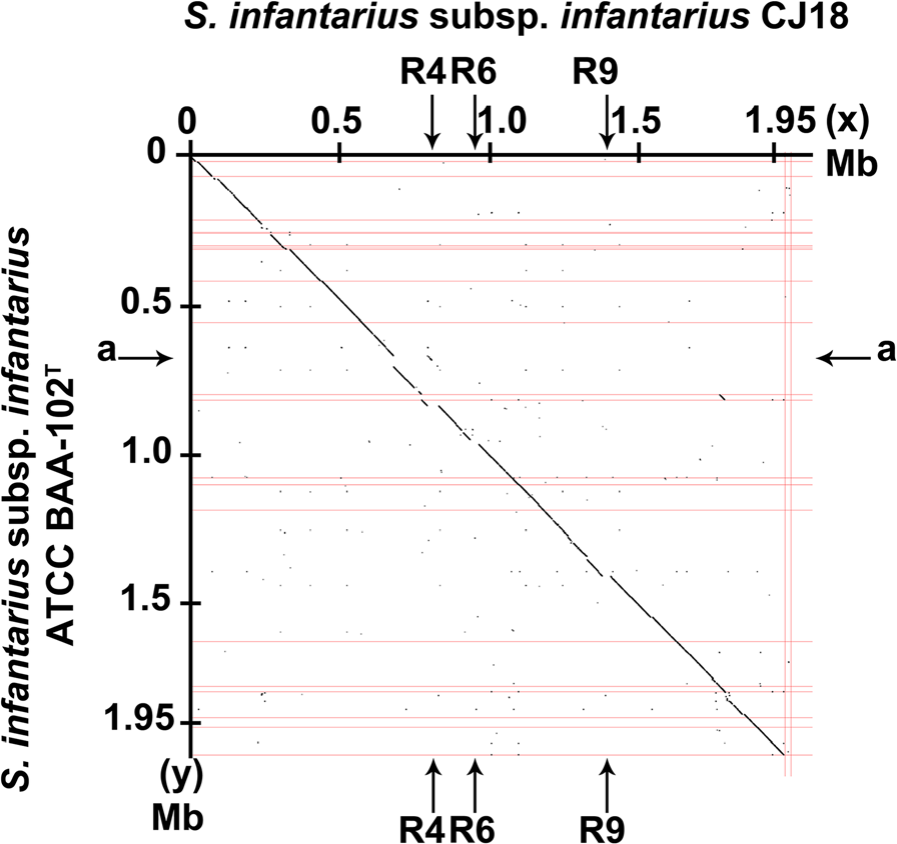
**Synteny plot of genomes *Sii *CJ18 (x) vs. *Sii *ATCC BAA-102**^**T **^**(y).** Both genomes of the *Sii* strains display a high degree of conservation indicated by the alignment near the diagonal line. Major insertion sites can be identified as R4 (34.2 kb) consisting largely of phage-related genes; R6 (25.6 kb) encompassing a 13.2-kb *S. thermophilus*-gene cluster comprising the additional *gal-lac* operon; and R9 (26.1 kb) containing among others an HTH-type transcriptional regulator Rgg, primosomal protein N’ (replication factor Y) – superfamily II helicase, an FtsK/SpoIIIE family protein and a conjugal transfer protein. The major gap α (34.6 kb) in ATCC BAA-102^T^ corresponds to a phage region.

**Figure 4:**
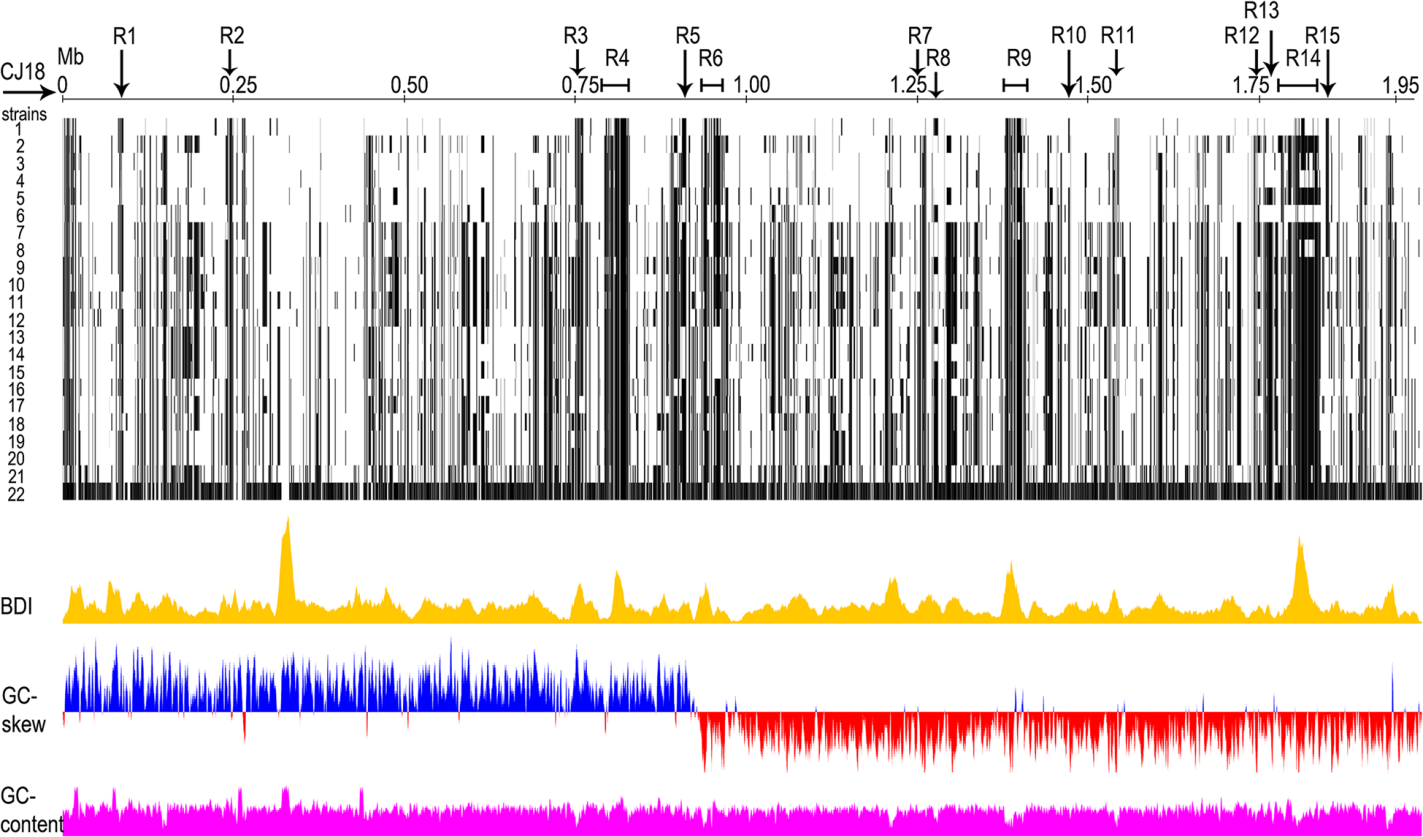
**Barcode plot of whole genome comparison of *Sii *CJ18 with genomes of related species.** Whole genomes of *Streptococcus* and *Lactococcus* strains were compared for the absence (black bar) and presence (white space) of certain genes related to those of CJ18. Several relevant regions (R1-15) were detected in CJ18 containing the following proteins: (R1) phage-related, (R2) cell-/environment signalling, (R3) Eps/Cps synthesis, (R4) phage-related, (R5) restriction endonuclease and methylase, (R6) *S. thermophilus*-related e.g. LacS/LacZ, (R7) metabolism, (R8) CRISPR-associated, (R9) hypothetical proteins unique for CJ18, (R10) *S. infantarius*-species-specific, (R11) surface antigen, (R12) putative bacteriocin locus inactive in CJ18, (R13) adhesion-related proteins, (R14) hypothetical proteins shared between *S. infantarius* and dairy *S. gallolyticus* subsp. *macedonicus* and (R15) 2^nd^ oligopeptide transport operon. Genome characteristic base-deviation index (BDI), GC-skew and GC-content of CJ18 are depicted below the comparison barcode chart. The strains used in this comparison are: 1.) *Sii* ATCC BAA-102^T^, 2.) *S. equinus* ATCC 9812, 3.) *S. gallolyticus* subsp. *gallolyticus* ATCC BAA-2069, 4.) *S. gallolyticus* subsp. *gallolyticus* UCN34, 5.) *S. bovis* ATCC 700338, 6.) *S. gallolyticus* subsp. *macedonicus* ACA-DC 198, 7.) *S. agalactiae* 2603 V/R, 8.) *S. agalactiae* NEM316, 9.) *S. equi* subsp. *equi* 4047, 10.) *S. dysgalactiae* subsp. *equisimilis* GGS_124, 11.) *S. pyogenes* M1 GAS, 12.) *S. equi* subsp. *zooepidemicus* MGCS10565, 13.) *S. thermophilus* LMG18311, 14.) *S. thermophilus* LMD-9, 15.) *S. thermophilus* CNRZ1066, 16.) *S. sanguinis* SK36, 17.) *S anginosus* 1_2_62CV, 18.) *S. mitis* B6, 19.) *S. pneumoniae* D39, 20.) *S. pneumoniae* R6, 21.) *Lc. lactis* subsp. *cremoris* MG1363 and 22.) artificial antibiotic resistance genome [37].

The draft genome sequence of the *Sii* ATCC BAA-102^T^ type strain was used for a comparison to the African isolate CJ18. An *in silico* hybridization revealed that the organization of loci was highly conserved between CJ18 and ATCC BAA-102^T^ (Figure 3) and to the closely related species *S. gallolyticus* subsp. *macedonicus* and *S. gallolyticus* subsp. *gallolyticus*, albeit at a lesser degree [Additional file 2]. The genome of CJ18 is 37 kb larger than that of ATCC BAA-102^T^ and harbors a number of variable regions and insertions compared to other streptococci, designated R1-R15 (Figure 4 and [Additional file 1]). The major variable regions comprise phage-related proteins (R4, 34.2 kb), proteins with high sequence identity to *S. thermophilus* (R6, 25.6 kb) and a cluster of metabolic and hypothetical proteins specific for CJ18 (R9, 26.1 kb). Interestingly, R14 comprises many hypothetical proteins shared to the largest extent with the Greek cheese isolate *S. gallolyticus* subsp. *macedonicus* ACA-DC 198 (R14, 52.5 kb) and second to ATCC BAA-102^T^. This suggests a closer relationship among these SBSEC strains compared to the other strains used in genome analysis and might possibly even be related to the dairy origin. Remarkably, variable regions often possess a distinct base-deviation index in CJ18, indicating recent evolutionary origin due to little advanced amelioration (Figure 4).

For 179 CDSs in CJ18, no homologous CDS were detected in ATCC BAA-102^T^. However, homologous CDS were detected in other streptococci for 103 of them, whereas for the other 76 CDS no significant hits were found in related strains [Additional file 1] (Table 2). The reverse comparison revealed 310 CDS from ATCC BAA-102^T^ without orthologous CDS in CJ18, 97 of which encoded for hypothetical proteins [Additional file 1]. This comparison of the African dairy isolate CJ18 to the type strain reveals a high similarity in gene content and organisation. However, there are some remarkable differences in gene content suggesting a distinct evolution of the two strains.

**Table 2:** GenBank accession numbers and reference sequence numbers of strains used in this study

**Species**	**Strain**	**Source**	**Genbank accession or reference sequence number**	**Reference**
artificial antibiotic resistance genome	various	gene sequences of published antibiotic resistance genes	none	[37]
*Lactococcus lactis* subsp. *cremoris*	MG1363	international prototype for LAB genetics; plasmid-free descendant of NCDO712, a cheese starter	[GenBank:NC_009004]	[38]
*S. agalactiae*	2603 V/R	human clinical specimen	[GenBank:NC_004116]	[34]
*S. agalactiae*	NEM316	human clinical specimen	[GenBank:NC_004368]	[39]
*S. anginosus*	1_2_62CV	human clinical specimen	[GenBank:NZ_ADME00000000]	HMP ^a)^
*S. bovis*	ATCC 700338	human clinical specimen, synovial fluid from knee	[GenBank:NZ_AEEL00000000]	HMP ^a)^
*S. dysgalactiae* subsp*. equisimilis*	GGS_124	human clinical specimen	[GenBank:AP010935]	[40]
*S. equi* subsp*. equi*	4047	horse clinical specimen	[GenBank:FM204883]	[41]
*S. equi* subsp*. zooepidemicus*	MGCS10565	human clinical specimen	[GenBank:CP001129]	[42]
*S. equinus*	ATCC 9812	human clinical specimen, gut	[GenBank: AEVB00000000]	HMP^a)^
*S. gallolyticus* subsp*. gallolyticus*	ATCC 43143	human clinical specimen, blood	[GenBank:AP012053]	[32]
*S. gallolyticus* subsp*. gallolyticus*	ATCC BAA-2069	human clinical specimen, blood	[GenBank:FR824043]	[33]
*S. gallolyticus* subsp*. gallolyticus*	UCN34	human clinical specimen, blood	[GenBank:FN597254]	[13]
*S. gallolyticus* subsp*. gallolyticus*	TX20005	human clinical specimen, heart	[GenBank:NZ_AEEM00000000]	HMP ^a)^
*S. gallolyticus* subsp*. macedonicus (=S. macedonicus)*	ACA-DC 198	Greek kasseri cheese, dairy isolate	[GenBank:HE613569] (genome) and [GenBank:HE613570] (plasmid pSMA198)	[30,43,44]
*S. gallolyticus* subsp*. pasteurianus*	ATCC 43144	human clinical specimen, blood	[GenBank:AP012054]	[32]
*S. infantarius* subsp*. infantarius*	ATCC BAA-102^T^ (isogenetic strain of CCUG 43820^T^)	human infant, feces	[GenBank: ABJK00000000]	HMP ^a)^
*S. infantarius* subsp*. infantarius*	CJ18	fermented camel milk *suusac*	[GenBank:CP003295] (genome) and [GenBank:CP003296] (plasmid pSICJ18-1)	this study and [2,7,31]
*S. infantarius* subsp*. infantarius*	LP90	dairy origin	[GenBank:HM008642]	none
*S. mitis*	B6	hospital isolate Germany	[GenBank:NC_013853]	[45]
*S. pneumoniae*	D39 (=NCTC 7466)	virulent human clinical isolate	[GenBank:NC_008533]	[36]
*S. pneumoniae*	R6 (=ATCC BAA-255)	unencapsulated, parent strain R36A derived from D39	[GenBank:NC_003098]	[46]
*S. pneumoniae*	TIGR4	human clinical isolate	[GenBank:NC_003028]	[47]
*S. pyogenes*	M1 GAS (=SF370)	human clinical isolate	[GenBank:NC_002737]	[35]
*S. salivarius*	ATCC 25975	human saliva	[GenBank:AF389474]	[48]
*S. sanguinis*	SK36	human dental plaque	[GenBank:NC_009009]	[49]
*S. thermophilus*	CNRZ1066	yogurt	[GenBank:NC_006449]	[26]
*S. thermophilus*	LMG18311	yogurt	[GenBank:NC_006448]	[26]
*S. thermophilus*	LMD-9 (=ATCC BAA-491)	yogurt	[GenBank:NC_008532]	[27]

^a)^*HMP* Human microbiome project http://www.hmpdacc.org.

### Carbohydrate metabolism

Carbohydrate transport in bacteria is frequently mediated via phosphotransferase systems (PTSs). PTS encoding operons were detected in both *Sii* strains for the uptake of β-glucosides, lactose, fructose/mannose, fructose, sucrose, maltose/glucose and cellobiose. Such a wide variety of transport systems is often observed in GI-tract associated microbes [50]. Remarkably, the lactose PTS gene locus in CJ18 (Sinf_0190-0195) is interrupted by three transposases, two truncating the β-glucoside Bgl operon antiterminator upstream of the PTS genes and one within the 6-phospho-β-galactosidase downstream of the PTS genes, suggesting that the lactose PTS in CJ18 is not involved anymore in lactose utilization.

Genes involved in galactose utilization in CJ18 are organized in the operon *galRKTE2* operon (Sinf_0205-0208). However, compared to ATCC BAA-102^T^, CJ18 harbors an additional *gal-lac* operon comprising genes *galT*(truncated)/*galE1M/lacSZ* (Sinf_0939-Sinf_0935) with high sequence identity (>91%) to *S. thermophilus*[7] and localized in region R6 [Additional file 3]. Also genes in the proximity of this *gal-lac* operon display high sequence identity to *S. thermophilus*, comprising among others the putative virulence gene encoding exfoliative toxin B (Sinf_0933), an acyl-CoA dehydrogenase (Sinf_0932) and a macrophage infectivity potentiator (Sinf_0931) [Additional file 1][Additional file 3]. Although the high sequence conservation indicates an *S. thermophilus* origin, the sequential order of genes is only conserved in the *gal-lac* operon. Mainly non-conserved DNA sequences were localized downstream of the *gal-lac* operon and the truncated *galT*.

Surprisingly, a second *lacS* (Sinf_1514) was detected in both *Sii* strains not adjacent to either the *gal* or *gal-lac* operon. This second LacS displays 98.9% amino acid sequence identity between the two *S. infantarius* strains and lower identity (60%) to the *S. thermophilus*-like LacS (Sinf_0936)*.* The physiological role of this second LacS is unknown.

**Figure 5:**
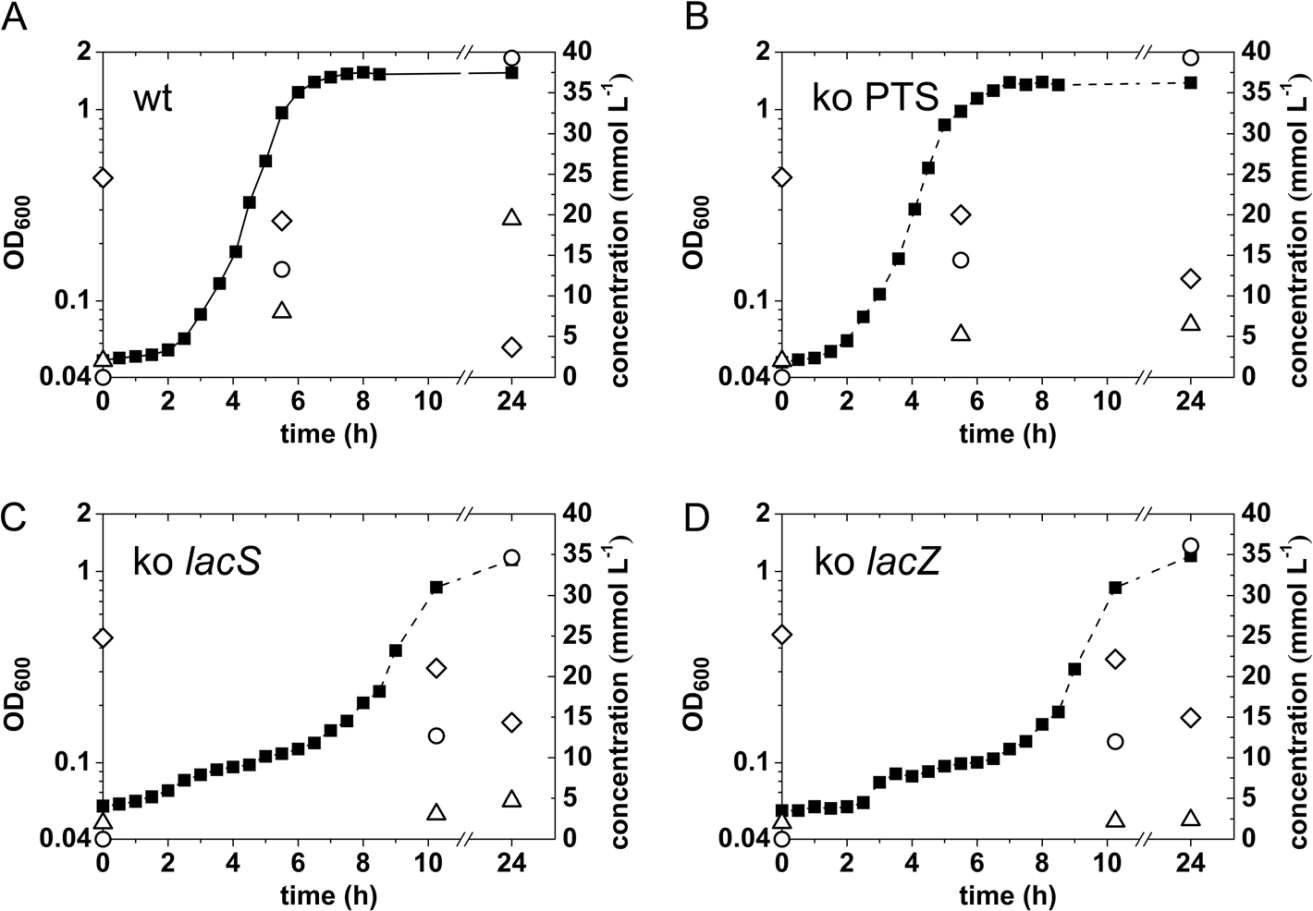
**Growth kinetics of wild type and knock-out (KO) strains of *Sii *CJ18 in lactose medium.** Growth kinetics of CJ18 wild type (**A**), CJ18Δ*lacIIC* (**B**), CJ18Δ*lacS* (**C**) and CJ18Δ*lacZ* (**D**) were compared in Elliker-based lactose medium for optical density (OD_600_ ■) and for metabolites lactose (◊),lactate (○) and galactose (ᐃ) in cell-free supernatant. Representative curves of two independent repetitions per strain are shown.

To elucidate the role of two lactose transport systems in lactose metabolism of CJ18, knock-out (KO) strains were constructed in the lactose translocater *lacS* (Sinf_0936), the β-galactosidase *lacZ* (Sinf_0935) and the permease unit of the lactose PTS encoding gene *lacIIC* (Sinf_0192) using a single-cross-over strategy (Table 3). Phenotypes of KO strains were confirmed on BHI/X-Gal/IPTG agar media yielding blue colonies for CJ18^WT^ (wild type), CJ18Δ*lacIIC*, CJ18Δ*lacS* and white colonies for CJ18Δ*lacZ.* This indicates no polar effects of *lacS* disruption on the expression of the *lacZ* gene downstream of *lacS* [Additional file 4]*.* The wild type CJ18 and its mutant derivatives CJ18Δ*lacIIC*, CJ18Δ*lacS* and CJ18Δ*lacZ* grew similarly in control medium containing glucose as sole carbon source [Additional file 5]. When grown with lactose as sole carbon source, CJ18Δ*lacIIC* displayed a similar growth pattern as the wild type CJ18 (Figure 5), indicating that lactose uptake in CJ18 is not mediated by the lactose PTS. Strains disrupted in genes of the *gal-lac* operon, CJ18Δ*lacS* and CJ18Δ*lacZ* had clearly an impaired growth rate on lactose (Figure 5). The growth characteristics of the mutant strains CJ18Δ*lacS* and CJ18Δ*lacZ* on lactose show that lactose is utilized in CJ18 via uptake by LacS and subsequently cleaved by LacZ with a similar mechanism to the lactose metabolism of *S. thermophilus.*

**Table 3:** Strains and plasmids used in this study

**Material**	**Relevant features**^**a**^	**Source**
**Strains**		
*Streptococcus infantarius* subsp*. infantarius*		
CJ18	Wild type strain, *suusac* isolate	[2,7,31]
CJ18/pVE6007	CJ18 derivative carrying pVE6007, Cm^R^	this study
CJ18Δ*lacIIC*	*lacIIC::pLFB1005*, *lacIIC* gene disruption derivative of CJ18, Em^R^	this study
CJ18Δ*lacZ*	*lacZ::pLFB1006*, *lacZ* gene disruption derivative of CJ18, Em^R^	this study
CJ18Δ*lacS*	*lacS::pLFB1007*, *lacS* gene disruption derivative of CJ18, Em^R^	this study
*Lactococcus lactis*		
LL302	RepA^+^ derivative of MG1363, host for pORI28	[51]
**Plasmids**		
pORI28	Em^R^, Ori^+^, RepA^-^, pWV01 derivative, vector for chromosomal insertions in Gram-positive bacteria	[52]
pVE6007	Cm^R^, thermosensitive derivative of pWV01, carrier plasmid for pORI28	[53]
pLFB1005	Em^R^, pORI28 derivative containing a 939-bp internal fragment of *lacIIC.*	this study
pLFB1006	Em^R^, pORI28 derivative containing an 1177-bp internal fragment of *lacZ.*	this study
pLFB1007	Em^R^, pORI28 derivative containing a 900-bp internal fragment of *lacS.*	this study

^a^*Cm*^*R*^ Chloramphenicol resistant; *Em*^*R*^ Erythromycin resistant.

### Additional features related to dairy environment

Oligopeptide transporters are important during growth in milk for the uptake of peptides and amino acids [54,55]. Similar to ATCC BAA-102^T^, CJ18 possesses an OppABCDF peptide transport system (Sinf_0305-0309) but the genome of CJ18 encodes two additional OppA (Sinf_1225 and Sinf_1226) and, remarkably, a second OppABCDF encoding operon (Sinf_1825-1821, region R15, Figure 4) with high sequence identity to *Streptococcus equi*, *Streptococcus pyogenes* or *Streptococcus gordonii* [Additional file 1]. Single amino acid transport systems are conserved in both strains and in contrast to *S. thermophilus* strains, no reduction in amino acid biosynthesis pathways was observed for CJ18. Both *S. infantarius* strains encode apparent complete pathways, such as histidine and glutamate biosynthesis or arginine catabolism (CJ18).

Capsular polysaccharides (CPS) and exopolysaccharides (EPS) are involved in the adhesion properties of bacteria through biofilm formation and serve as a defense mechanism against immune responses [56,57]. Furthermore, EPS may contribute to the texture of many dairy products. CJ18 and ATCC BAA-102^T^ both possess a conserved 5-kb operon for EPS biosynthesis. The genetic organization downstream of this cluster differs between the two *S. infantarius* strains. CJ18 harbors a number of additional EPS and CPS biosynthesis genes (R3, Figure 4) that share highest protein sequence identities with proteins of species outside of the SBSEC. Remarkably, the same region in CJ18 contains *wefC* encoding a receptor polysaccharide phosphotransferase, also termed stealth protein. This gene is absent in ATCC BAA-102^T^ and displays high sequence homology to CpsJ of *S. thermophilus* (99%). Based on *in silico* analysis it was hypothesized to be involved in protection from the host immune system [58]. The presence of a high variety of EPS genes could be caused by selection during *suusac* manufacturing, but could also imply an additional virulence risk if a strain displays further virulence factors for e.g. invasion, infection or toxin production.

### Adhesion and other virulence factors

Adhesion of bacteria to surfaces is influenced by many factors such as EPS or CPS production as mentioned above, but also certain specific proteins. A fibronectin binding protein Fpb involved with adhesion to fibronectin and fibrinogen is present in both ATCC BAA-102^T^ and CJ18. *Streptococcus bovis* group surface proteins (Sbs) are also involved in adhesion and found in both CJ18 (7 genes) and in ATCC BAA-102^T^ (8 genes). Five of these Sbs are organized in a 13.7-kb region (R13, Figure 4) in CJ18 comprising a truncated Sbs 13 (collagen binding protein, Sinf_1737), an LPXTG-specific A/C-type sortase (Sinf_1742), Sbs14 (autotransporter adhesion/cell wall anchored protein, Sinf_1743) and Sbs15 (ribonuclease G and E/peptidoglycan linked protein, Sinf_1744). This region upstream of Sbs 13 is conserved in CJ18 and ATCC BAA-102^T^, the dairy isolate *S. gallolyticus* subsp. *macedonicus* ACA-DC 198 and other *S. gallolyticus* strains. The presence of Sbs4 and Sbs9 suggests that certain adhesion factors are shared among SBSEC as commensal inhabitants of gastrointestinal tracts and detected also in the dairy strain *S. thermophilus* LMG18311. These factors might only contribute to virulence if further factors for invasion or toxin production are present as well. Other adhesion factors like *S. bovis* adhesion proteins (Acb) or others from non-SBSEC origin, such as FimA and FimB, are not present in both *Sii* strains.

A hemolysin III protein highly identical to that of the *S. gallolyticus* group including *S. gallolyticus* subsp. *macedonicus* ACA-DC 198 (91%) as well as that of *S. thermophilus* LMD-9 (80%) is encoded in both *Sii* strains. No defibrinated sheep blood hemolysing activity was detected for both strains. A direct implication of virulence from the presence of a hemolysin gene except streptolysin O is not yet established for streptococci [59].

Typical virulence factors of non-SBSEC-members *S. pyogenes*, *S. agalactiae* and *S. pneumoniae* had been used for the safety evaluation of *S. thermophilus*[26]. Some of these virulence factors were previously found in *S. gallolyticus* UCN34 such as *ssaB/scaA/psaA* (locus tag Gallo_2047)*, pilB* (Gallo_0087)*, gtfbC* (Gallo_1055)*, atlA* (Gallo_1368) [32] and used to screen strains in this study. *ssaB/scaA/psaA* was not detected in *Sii* strains whereas *atlA* displayed a lower protein sequence identity in CJ18 (49%) compared to the cheese isolate *S. gallolyticus* subsp. *macedonicus* ACA-DC 198 (91%). Pro-inflammatory proteins [23] were detected in both *Sii* strains but also in *S. thermophilus* since they encode basic metabolic functions. Finally, comparison with an *in silico* genome containing antimicrobial resistance and virulence factor genes [37,60] did not result in significant hits with any typical or concerning streptococcal virulence factors for both CJ18 and ATCC BAA-102^T^.

### Natural competence

Several regions potentially involved in natural competence were detected in both *S. infantarius* strains. These include a competence operon (*comGA/GB/GC/GD/GE/GF/GG*), separate competence genes and a CoiA encoding gene involved in DNA uptake. Furthermore, a CJ18-unique restriction endonuclease and methylase were detected in region R5 (Figure 4). In addition, both strains contain recombination proteins like RecA, the Rossman fold nucleotide-binding protein Smf/DprA and the single-strand DNA binding protein SsbB [61,62]. The organization and mechanism of the competence-related genes (*comX/sigX* and *comS* promoters) seems to be conserved in both ATCC BAA-102^T^ and CJ18 as well as most other streptococci [63]. However, CJ18 harbors an additional conjugal transfer protein (Sinf_1366 region R9, Figure 3) with high protein sequence identity (82%) to *S. thermophilus* variant, suggesting a potentially increased capability for DNA uptake compared to ATCC BAA-102^T^.

This is further supported by the apparent reduced activity of Clustered Regularly Interspaced Short Palindromic Repeats (CRISPRs) and CRISPR associated genes (*cas*) forming the CRISPR/Cas system for defense against foreign DNA [64]. Both CJ18 and ATCC BAA-102^T^ harbor single copies of *csn2*, *cas1* and *cas2* in region R8 (Figure 4). But remarkably, the CJ18 proteins Csn2, Cas1 and Cas2 had higher identity (88-93%) with the corresponding proteins in *S. gallolyticus*, *S. bovis* and *S. equinus* than with ATCC BAA-102^T^. A CRISPR array comprises a leader sequence followed by identical repeated DNA sequences intersected by highly variable spacer sequences. CJ18 comprises a CRISPR/Cas section with 9 spacers whereas ATCC BAA-102^T^ harbors 29 spacers. The relative low number of CRISPR spacers predicts a lower CRISPR activity in CJ18 and thus a decreased protection against foreign DNA.

No DNA sequence identity was detected between any of the spacers. This indicates strain dependent Cas/CRISPR activity in *S. infantarius* also reported for *S. thermophilus* strains [65].

### Other features in the CJ18 genome

Production of bacteriocins is widely distributed among streptococci [66]. *S. infantarius* CJ18, ATCC BAA-102^T^ and also LP90 (Table 2) possess a highly conserved bacteriocin ABC-transporter accessory protein InfAE-acc, shared also with *S. gallolyticus* strains (competence-stimulating peptide ABC transporter-permease ComB Sinf_1732) and the bacteriocin ABC-transporter InfAE-ABC (competence-stimulating peptide ABC transporter ATP-binding protein ComA Sinf_1731) located in region R12. Putative bacteriocin encoding genes were detected in ATCC BAA-102^T^, but none in strain CJ18, which confirms previous findings on its inability to produce bacteriocin-like inhibitory substances [2].

Unique phage-related genes are located in CJ18 in regions R1 and R4 (Figure 4). CJ18 and ATCC BAA102^T^ harbor both four and five phage integrase genes, respectively. However, only one of them (Sinf_0428) has a homologous gene in ATCC BAA-102^T^ (100% nucleotide identity), indicating possible distant relationship between these strains.

Remarkable differences between both *S. infantarius* strains and their closest related species within the SBSEC *S. gallolyticus* UCN34 (Figure 2) were a reduction in carbohydrate transport systems, e.g. the absence of trehalose and mannitol transporting and degrading enzymes which play a role in maintenance in the bovine rumen. This indicates a generally lower adaptation of *S. infantarius* to the bovine rumen as a habitat compared to *S. gallolyticus* and provides additional evidence to separate both species from each other.

## Discussion

Fermented dairy products are important in Africa as source of nutrients and as weaning food. Fermentation is an essential preservation method in the absence of refrigeration [67-69]. Analyses of dairy adaptations and potential virulence factors of bacteria leading spontaneous fermentation processes is therefore important to identify consumers’ health risk potential and unravel novel fermentative lactic acid bacteria strains.

In this study, we report the complete genome sequence of the African dairy isolate *Sii* CJ18, the first complete assembled genome of a *S. infantarius* species. Whole genome comparison of *Sii* CJ18 to *Sii* ATCC BAA-102^T^ and related streptococci revealed substantial adaptations to the dairy environment in CJ18, paralleling that of *S. thermophilus*. However, our data indicates that genome decay of *Sii* CJ18 is in a less advanced state compared to *S. thermophilus*, since most biosynthesis pathways seem to be intact and the number of pseudogenes (4.9%) is smaller than for *S. thermophilus* (10-19%). This suggests that establishment of CJ18 in the dairy environment is more recent than *S. thermophilus* strains or *S. gallolyticus* subsp*. macedonicus* ACA-DC 198*.* Based on genome decay, the most recent common ancestor for *S. thermophilus* strains was estimated to have lived 3,000-30,000 years ago, which is approximately the duration of human dairy activity [26,70]. Camels, however, were introduced in East Africa only around 2,500 years ago [71-73], and the less advanced state of genome decay in CJ18 may be related to the later start of African camel milk fermentation.

Adaptation to the dairy environment in *S. thermophilus* consists of enhanced uptake of lactose and peptides and loss of other metabolic pathways. CJ18 displays a similar adaptation in the lactose metabolism through the transporter LacS and β-galactosidase LacZ. Truncation of either LacS or LacZ resulted in significant impaired growth on lactose, confirming the functionality of this acquired lactose utilization path. Neither the second LacS (Sinf_1514), present in both CJ18 and ATCC BAA-102^T^, nor the lactose PTS could take over lactose transport in the LacS KO strain. The integration of transposases in the corresponding lactose PTS gene cluster seems therefore a result of loss of essentiality after the acquirement of *lacS* and *lacZ*. Moreover, a concurrent activity of both transporters potentially leads to misbalance in redox or phosphorylation status of the cell, and hence positive selection on truncation of the lactose PTS gene cluster might have even occurred after acquirement of LacSZ*.* The release of galactose into the growth medium shows that LacS in CJ18 functions as a highly efficient antiporter and the competitiveness of CJ18 in the dairy environment seems therefore based on the acquired LacSZ. This facilitates efficient transport of lactose and as a consequence an increased lactose consumption and lactate production compared to ATCC BAA-102^T^ (isogenetic strain of CCUG 43820^T^) [7].

The role of other adaptations to the dairy environment, such as the presence of a second *oppABCDF* operon and an extended EPS biosynthesis cluster is less clear. Enhanced uptake of casein derived peptides by the second peptide transporter could contribute to increased competiveness in milk. The enlarged cluster of Eps/Cps-related proteins could contribute to survival during the *suusac* back-slopping process, via improved biofilm formation capabilities. Furthermore, EPS contribute to texture of the fermented dairy product and the selection of strains for these textural properties might have occurred in the past [18,26].

The more recent adaptation to the dairy environment of C18 is reflected by the lower number of pseudogenes and CRISPR spacers in CJ18 compared to *S. thermophilus* or *S. gallolyticus* subsp. *macedonicus* ACA-DC 198. CJ18 harbors nine CRISPR spacers whereas typical widespread dairy starter strains of *S. thermophilus* such as CNRZ 1066 and LMG 18311 harbor 42 and 39 spacers, respectively [26,65]. Phage infection and phage-related fermentation losses are major problems in dairy technology. The number of CRISPR spacer in a bacterial genome is directly linked to phage contact history and presumptive resistance against phages of that particular strain [74]. The African strain CJ18 was apparently not continuously exposed to phage infections over prolonged periods. This could be a result of the spontaneous nature of the traditional fermentation, which in contrast to industrial starter culture fermentations, does not rely on selected starter strains. The absence of CRISPR spacer identity between CJ18 and ATCC BAA-102^T^ further shows that the African CJ18 is only a distant relative of ATCC BAA-102^T^ as previously observed in microevolution of CRISPR spacers in other genera [75]. Additionally, the presence of 103 CDS in CJ18 shared only with other streptococci but not with ATCC BAA-102^T^ as well as the absence of 310 CDS in CJ18 present in ATCC BAA-102^T^ indicates an ancestral streptococcal origin of these CDSs and again only distant relation between the two *Sii* strains.

Another interesting feature of CJ18 is its natural competence and DNA uptake capability, paralleling that of other streptococci and lactic acid bacteria (LAB) [27,76]. As a possible result of this, the genome displays traces of HGT events from commensal bacteria encountered in milk such as *Lactococcus* spp. and *S. thermophilus* but also pathogens like *S. agalactiae*. Furthermore, the natural competence could potentially contribute to the uptake of mobile genetic elements and to spread of antibiotic resistance genes [2]. Therefore the apparent intact competence machinery is probably of high importance for persistence of the strain in the African dairy environment.

CJ18 harbors none of the concerning typical streptococcal virulence factors [60] and less SBSEC-related virulence factors compared to e.g. *S. gallolyticus* and *S. bovis*. Moreover, most of these potential virulence factors are related to adhesion and not directly to infection, cytotoxicity or toxin production and are therefore of less concern. Many factors found in CJ18 are also present in the proclaimed safe strain *S. gallolyticus* subsp. *macedonicus* ACA-DC 198, a species without QPS-approval [25,77]. Some potential virulence factors or artifacts thereof were even found in *S. thermophilus*. Consequently, relying on genomic information alone, ingestion and digestion of large amounts of *Sii* via *suusac* does not seem to be a direct health risk for adults. However, the SBSEC-associated health risks for immune-deprived people, a major concern in Africa, and for children are less understood as epidemiological data on these diseases are not available. Furthermore, the uncertain association of *Sii* with human diseases necessitates further elucidation of presumptive *Sii*-specific virulence factors or the absence thereof in *Sii*.

## Conclusions

We assembled and analyzed the first complete genome sequence of the species *S. infantarius*. The African dairy strain *Sii* CJ18 revealed many genetic adaptations to the dairy environment through acquired carbohydrate utilization pathways resulting in a lactose metabolism paralleling that of *S. thermophilus*. Potential mutations and insertions resulting in pseudogenes or truncated gene clusters indicate further evolution paralleling *S. thermophilus*. However, gene decay is not as advanced as in the dairy isolates *S. thermophilus* or *S. gallolyticus* subsp*. macedonicus* ACA-DC 198 and the establishment in the dairy environment is therefore likely from a younger evolutionary period.

The species *S. infantarius* harbors less virulence factors compared to the *S. gallolyticus* group. However, specific virulence factors for *S. infantarius* are not yet identified and epidemiological studies are necessary to prove the innocuity of African dairy *Sii* strains and milks predominantly fermented with these strains. This could prove traditional dairy fermentation in Africa as ideal process to enhance food safety and shelf life as well as the later application of *Sii* in an enhanced traditional fermentation technology paralleling the Western dairy industry, but specific for Africa. Conclusively, this study provides insight into the evolution of a novel dairy species and dairy environment in parallel to the Western counterpart.

## Methods

### Bacterial strains and culture conditions

Strains and plasmids used in this study are listed in Table 3. *Lactococcus lactis* LL302 was used as intermediate cloning host and cultured without agitation at 30°C in M17 (Biolife, Milan, Italy) [78], supplemented with 0.5% glucose (G-M17). *Sii* strains were grown overnight in G-M17 at 37°C for production of pre-cultures, or anaerobically on G-M17 plates at 37°C.

For growth profiling on specific carbohydrates, a pre-culture of *Sii* in G-M17 was used to inoculate (1% v/v) Elliker-based single carbohydrate medium [79,80], containing either glucose (1%) or lactose (1%). Growth profiling was performed in 125-mL butyl-rubber stoppered serum flasks [7] at 37°C for the determination of growth curves.

When appropriate, chloramphenicol and erythromycin were added to the media at a final concentration of 8 μg mL^-1^ and 10 μg mL^-1^, respectively. BHI agar media (Biolife) supplemented with 80 mg mL^-1^ 5-bromo-4-chlor-3-indolyl-b-D-galactopyranoside (X-Gal, AppliChem, Darmstadt, Germany) and 0.5 mM isopropyl-b-D-thiogalactopyranosid (IPTG, AppliChem) was used to confirm phenotypes of KO strains. AnaeroGen packs (Oxoid, Pratteln, Switzerland) were used as oxygen scavengers for agar plate incubation in anaerobic jars. Stock cultures of all strains were stored at −80°C in 30% glycerol (v/v). All chemicals and enzymes used in this study were obtained from Sigma-Aldrich (Buchs, Switzerland), unless stated otherwise.

### Genbank and reference sequence accession numbers

The genome sequence and plasmid pSICJ18-1 of *Sii* CJ18 is available in the nucleotide database GenBank under the accession numbers [GenBank: CP003295, GenBank: CP003296] [31]. A summary of GenBank accession and reference sequence numbers of strains used in this study for bioinformatic analyses are provided in Table 2.

### Electroporation of *Sii* CJ18 and *Lactococcus lactis* LL302

*Lc. lactis* LL302 and *Sii* strains were transformed by electroporation using a procedure developed for *Lc. lactis*[81]. Positive transformants were selected on G-M17 agar media supplemented with chloramphenicol (8 μg mL^-1^) or erythromycin (10 μg mL^-1^) as required after aerobic incubation at 30°C for 1–2 days.

### DNA manipulations

Molecular cloning and DNA manipulations were essentially performed as described by Sambrook et al. [82]. Plasmid DNA isolation from *Lc. lactis* LL302 was performed using an alkali cell lysis method after lysozyme treatment with subsequent purification [83] using a Midiprep Kit (Qiagen, Basel, Switzerland). Restriction enzymes and Phusion-polymerase were obtained from New England Biolabs (Frankfurt am Main, Germany) and T4-ligase from Invitrogen (Basel, Switzerland). Primers were purchased from Microsynth (Balgach, Switzerland).

### Construction of mutant strains

For inactivation of the lactose PTS, the permease encoding *lacIIBC* gene (Sinf_0192) was disrupted using a single-cross-over strategy. A 959-bp internal fragment of *lacIIC* was amplified using a PCR master mix (Thermo Scientific, St. Leon-Rot, Germany), chromosomal DNA of CJ18 as template and the primers lacIIC_for and lacIIC_rev (Table 4). The obtained product was purified using a GFX purification column (GE Healthcare, Glattbrugg, Switzerland) and digested with *BamH*I and *EcoR*I (restriction sites introduced in primers). The restricted fragment (939 bp) was cloned into a *BamH*I/*EcoR*I digested pORI28 resulting in pLFB1005, a *lacIIBC* disruption vector. Similarly, a 900-bp internal fragment of the lactose transporter gene *lacS* was amplified using primers lacS_for and lacS_rev. The product was purified, restricted with *BamH*I and *EcoR*I, and cloned into a *BamH*I/*EcoR*I digested pORI28, resulting in pLFB1007, a *lacS* interrupting vector.

**Table 4:** Oligonucleotides used to amplify internal fragments of target genes to construct knock-out strains

**Name**	**Sequence (5’ to 3’)**^**a**^	**Name**	**Sequence (5’ to 3’)**
lacS_for	GATCGGATCCGATCCAAAGCAAAATAGTCA	lacS_con_for	TCCTATGCAGCGGGTGCTT
lacS_rev	GATCGAATTCTGCAGTCAAGATAATTGGA	lacS_con_rev	GAGATAATCATAAGGATAACAA
lacZ_for	GATCCTGCAGGCGTTAATACAGTTGACGCTCAC	lacZ_con_for	TTACTTAAACGATCCAAAGA
lacZ_rev	GATCGGATCCTTTGCCATGTACCGTGTGTT	lacZ_con_rev	CATGTTATTGGCACGATCCA
lacIIC_for	GATCGGATCCAATATTTGCGAGCGATTCGT	lacIIC_con_for	GGAAACCATTCTTTGAGAG
lacIIC_rev	GATCGAATTCTACAATTGGAGCACCGAACA	lacIIC_con_rev	ATTTGAAGATCCACACGTT
pORI_for	TTG ATA ATG AAC TGT GCT GA	pORI _rev	ACG AAT CGC CAA CGT TTT CG

^a)^ Endonuclease restriction sites introduced in primers are underlined.

For disruption of *lacZ*, an 1177-bp internal fragment was amplified using primers lacZ_for and lacZ_rev. The product was digested with *BamH*I and *Pst*I and cloned into a similar digested pORI28, resulting in pLFB1006, a disruption vector for *lacZ*.

The obtained plasmids were first transformed into *Lc. lactis* LL302 for multiplication. After extraction, they were transformed into *Sii* CJ18 harboring the thermosensitive plasmid pVE6007 (Cm^R^) as carrier plasmid for pORI28 derivatives (Em^R^, Table 3). Transformants were isolated on G-M17 supplemented with 10 μg ml^-1^ erythromycin at 30°C. Growth of transformants at 37°C results in loss of pVE6007 and pORI28-derivatives cannot replicate anymore in the cells, forcing the plasmids to integrate into the chromosome. Therefore, colonies were picked, the presence of the correct plasmids confirmed by PCR and subsequently grown at 37°C in G-M17 supplemented with erythromycin for 24 h. Primary integrants were then isolated on G-M17 supplemented with erythromycin. To check for the loss of pVE6007, colonies were picked and transferred to G-M17 plates with 10 μg mL^-1^ chloramphenicol and grown overnight at 30°C. Colonies displaying an erythromycin resistant and chloramphenicol sensitive phenotype were checked for correct integration by PCR, using primers annealing outside of the region of integration in the chromosome (control primers in Table 4) and primers annealing in pORI28 (pORI28_for and pORI28_rev). Integrants showing the correct phenotype and positive PCR analyses were streaked on G-M17 with erythromycin and a single colony isolate was checked again by PCR. Phenotypes of KO strains were confirmed using BHI/X-Gal/IPTG agar media.

### Metabolite analysis by HPLC

Carbohydrate metabolites lactose, glucose, galactose, lactate and acetate were analyzed from bacterial culture supernatants on a Merck Hitachi HPLC system (Merck Hitachi, Darmstadt, Germany) as previously described [7].

### Genome annotation

DNA isolation, sequencing and assembly of the genome of CJ18 was previously described [31]. Annotation of the assembled *Sii* CJ18 and metabolic reconstruction was performed on the RAST server [84]. The primary gene annotation by RAST was verified by comparing each RAST-predicted gene to the annotated genes of the species listed in Table 1. The genes were categorized into four groups: correct, possible frameshift, possible wrong start/stop assignment and non-conserved hypothetical. Each gene predicted by RAST plus 60-bp flanking regions were translated *in silico* and the three possible reading frames were compared to all annotated genes within genomes of related species (Table 1) using the Smith-Waterman algorithm [85] on the basis of the BLOSUM62 substitution matrix. The score of the best match was compared to the self-alignment score of the original gene. If the highest score/self-alignment score-ratio was above 0.6, the gene was categorized as correct. If one of the two alternative reading frames had a score ratio above 0.75, the gene was assigned as having a possible frameshift. If the original gene was aligned to its best match with the number of either starting or ending gaps of more than 20-bp, it was categorized as possible wrong start/stop assignment. Genes with highest score/self-alignment score-ratios below 0.35, or a Needleman-Wunsch-Alignment to its best match with a negative score, were assigned as non-conserved hypothetical. The prediction of the oriC region upstream of *dnaA* was performed using Ori-finder [86].

### Phylogenetic analyses

DNA sequences were retrieved from GenBank or sequenced in this study (Table 2). The following genes were used: *groEL, gyrB, recA, recN, rpoB, secA, secY, sodA* and 16S rRNA encoding genes.

Sequences were aligned in MEGA4.0 [87] using the ClustalW algorithm and then trimmed to equal lengths. Construction of phylogenetic trees was performed in MEGA4.0 using the Neighbor-Joining method and a bootstrap test with 1000 repetitions followed by the computation of evolutionary distances using the Maximum Composite Likelihood method [87-90]. The resulting trees were rooted using *Lactococcus lactis* subsp*. cremoris* MG1363 as outgroup.

### Genome comparison – synteny plots

The raw scores for the local alignment of all putative proteins of *Sii* CJ18 versus all proteins of the strains of interest (Table 2) were calculated using the Smith-Waterman algorithm [85] on the basis of the BLOSUM62 substitution matrix [91]. The score ratio is calculated by dividing the raw score by the score of the protein of interest aligned to itself. A threshold of 0.4 was used to distinguish between similar and non-similar proteins [92]. A synteny plot was created by plotting the genomic location of all proteins of *Sii* CJ18 on the X-axis and the genomic location of all similar proteins of the strain of interest on the Y-axis.

The available contigs of *Sii* ATCC BAA-102^T^ were putatively assembled using Projector 2 [93]. The contigs of *Sii* ATCC BAA-102^T^ were re-annotated through the RAST pipeline to facilitate highest comparability with the genome of CJ18 annotated also via the RAST pipeline [84].

### Construction of genome comparison graphs

The Base Deviation Index (BDI) is calculated as the deviation of the base composition in a sliding 10-kb window to the average base composition over the entire genome using the *X*^*2*^ statistics [94]. The GC skew is calculated as $\frac{G-C}{G+C}$ with G and C being the number of guanin and cytosin in a sliding 1-kb window. The GC content is calculated as the percentage of guanin and cytosin in a sliding 1-kb window. Circular genome graphs were created using DNA Plotter [95].

### Search for bacteriocins

The genomic sequence of *Sii* CJ18 was translated *in silico* in all three possible reading frames. All peptides available in the BAGEL2-Bacteriocin-Database [96] were searched in the translated sequences using the Smith-Waterman algorithm [85] on the basis of the BLOSUM62 substitution matrix [91]. High scoring matches were further evaluated by hand.

### CRISPR/Cas analysis

CRISPRs were detected in the genomes of *Sii* CJ18 and ATCC BAA-102^T^ using CRISPRfinder and CRISPRdb [97,98]. Spacer sequences were aligned in BioEdit [99] through ClustalW after which DNA sequence identities were calculated. Amino acid sequences of CRISPR-associated (cas) proteins were analyzed analogous.

## Abbreviations

CDS: Coding DNA sequence; CPS: Capsular polysaccharides; CRISPR: Clustered regularly interspaced short palindromic repeats; EPS: Exopolysaccharides; KO: Knock-out; LAB: Lactic acid bacteria; PTS: Phosphotransferase system; QPS: Qualified presumption of safety; SBSEC: *Streptococcus bovis/Streptococcus equinus* complex; Sii: *Streptococcus infantarius* subsp. *infantarius.*

## Competing interests

The authors declare that they have no competing interests.

## Authors’ contributions

CJ performed genome assembly and analysis; RF performed bioinformatic analysis; MH performed construction of knock-out strains and experiments; CL and LM initiated and supervised the project; MJAS designed and supervised experiments and CJ, MJAS, LM and CL wrote/revised the paper. All authors have read and approved the final manuscript.

## Supplementary Material

Additional file 1 **Complete table of CDS in *Sii *genome CJ18 in comparison with reference strain genomes.** Description of data: This table contains all CDS of the African *Sii* genome CJ18 (excluding the plasmid) in comparison with reference strain genomes used in this study. A reverse comparison of CDS present in *Sii* ATCC BAA-102^T^ versus CJ18 is included. The CDS of the plasmid pSICJ18-1 are included in a separate tab with their highest NCBI Blast database gene match. Click here for file

Additional file 2 **Synteni plot of genome *Sii *CJ18 (x) vs. (A) *S. gallolyticus *subsp. *macedonicus *ACA-DC 198 (y), (B) *S. gallolyticus *subsp. *gallolyticus *UCN34 (y) and (C) *S. gallolyticus *subsp. *gallolyticus *ATCC BAA-2069 (y).** Description of data: Similar to the genome of *Sii* ATCC BAA-102^T^, *Sii* CJ18 and *S. gallolyticus* strains ACA-DC 198, ATCC BAA-2069 and UCN34 display a high degree of conservation indicated by the alignment near the diagonal line. The same major insertion sites as in ATCC BAA-102^T^ can be identified as R4 (34.2 kb) consisting largely of phage-related genes; R6 (25.6 kb) encompassing a 13.2-kb *S. thermophilus*-gene cluster comprising the additional *gal-lac* operon; and R9 (26.1 kb) containing among others an HTH-type transcriptional regulator rgg, primosomal protein N’ (replication factor Y) – superfamily II helicase and an FtsK/SpoIIIE family protein. The dairy isolate *S. gallolyticus* subsp. *macedonicus* ACA-DC 198 features an additional unique region R12 comprising bacteriocin-related structures of macedocin, salavaricin, lantibiotic modifying enzymes and transporters. Click here for file

Additional file 3 **Unique 13.2-kb gene locus with high DNA sequence identity to *S. thermophilus *in *Sii *genome CJ18.** Description of data: The African *Sii* CJ18 harbors an approximately 13.2-kb insert of DNA with high sequence identity to *S. thermophilus* LMD-9 (white arrows) within a 25.6-kb insert (R6, Sinf_0915-Sinf_0939). The 18.4-kb gap between Sinf_0910 to Sinf_0928 was largely occupied with hypothetical proteins of unknown origin, few transporters and phage-related genes. Genes are not drawn to scale. Gene numbering corresponds to CDS region Sinf_0910-Sinf_0938. Black arrows indicate *S. infantarius* identity; grey other streptococci and white *S. thermophilus* identity. Click here for file

Additional file 4 **Phenotypes of CJ18**^**WT **^**and mutant KO derivatives on BHI/X-Gal/IPTG agar media.** Description of data: Confirmation of phenotypes of wild type, reference and KO strains on BHI/X-Gal/IPTG agar media yielding blue colonies for CJ18^WT^, CJ18Δ*lacIIC*, CJ18Δ*lacS* and white colonies for CJ18Δ*lacZ* and CCUG 43820^T^. Click here for file

Additional file 5 **Growth kinetics of wild type and knock-out strains of *Sii *CJ18 in glucose medium.** Description of data: Growth kinetics of CJ18 wild type (A), CJ18Δ*lacIIC* (B), CJ18Δ*lacS* (C) and CJ18Δ*lacZ* (D) were compared in Elliker-based glucose medium for optical density (OD_600_ ■) and for metabolites glucose (∇),lactate (○) and galactose (ᐃ) in cell-free supernatant. Representative curves of two independent repetitions per strain are shown. Click here for file
