# Supplementary figures and images for: Comparative genome analysis of Streptococcus infantarius subsp. infantarius CJ18, an African fermented camel milk isolate with adaptations to dairy environment
Source: BMC Genomics. 2013 Mar 22;14:200. doi: 10.1186/1471-2164-14-200 (PMC3640971; doi:10.1186/1471-2164-14-200)

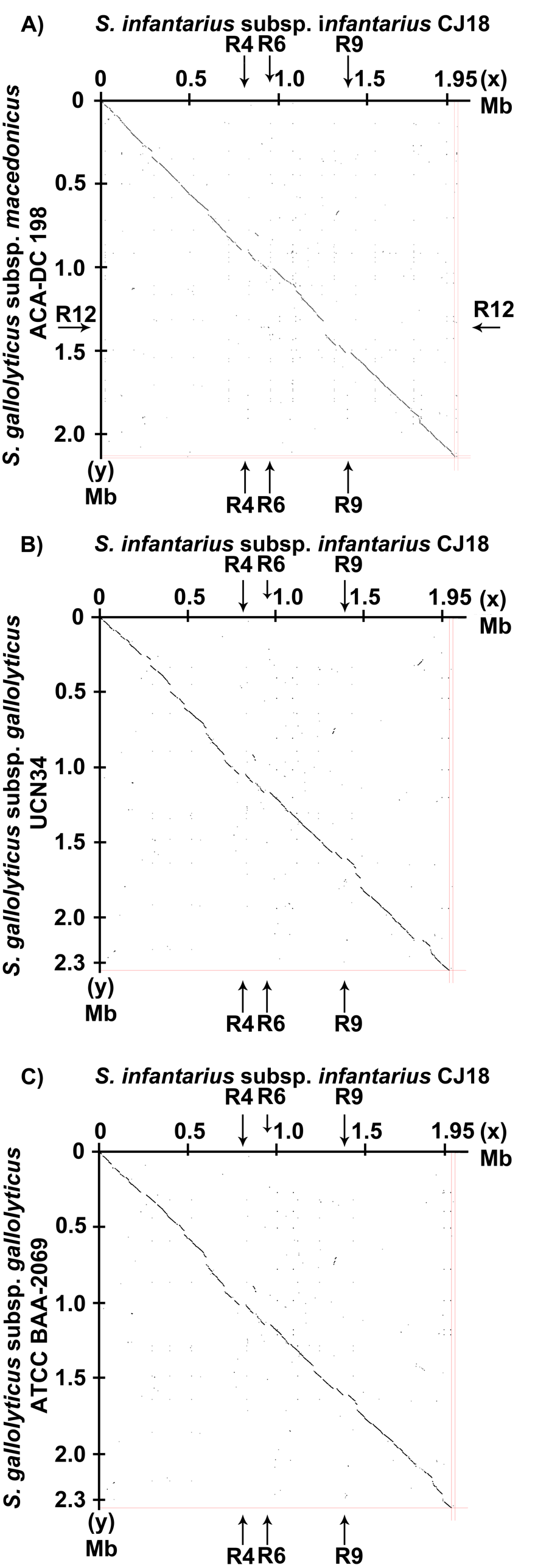

Supplement: Additional file 2 — Synteni plot of genome Sii CJ18 (x) vs. (A) S. gallolyticus subsp. macedonicus ACA-DC 198 (y), (B) S. gallolyticus subsp. gallolyticus UCN34 (y) and (C) S. gallolyticus subsp. gallolyticus ATCC BAA-2069 (y). Description of data: Similar to the genome of Sii ATCC BAA-102T, Sii CJ18 and S. gallolyticus strains ACA-DC 198, ATCC BAA-2069 and UCN34 display a high degree of conservation indicated by the alignment near the diagonal line. The same major insertion sites as in ATCC BAA-102T can be identified as R4 (34.2 kb) consisting largely of phage-related genes; R6 (25.6 kb) encompassing a 13.2-kb S. thermophilus-gene cluster comprising the additional gal-lac operon; and R9 (26.1 kb) containing among others an HTH-type transcriptional regulator rgg, primosomal protein N’ (replication factor Y) – superfamily II helicase and an FtsK/SpoIIIE family protein. The dairy isolate S. gallolyticus subsp. macedonicus ACA-DC 198 features an additional unique region R12 comprising bacteriocin-related structures of macedocin, salavaricin, lantibiotic modifying enzymes and transporters. [file 1471-2164-14-200-S2.tiff]

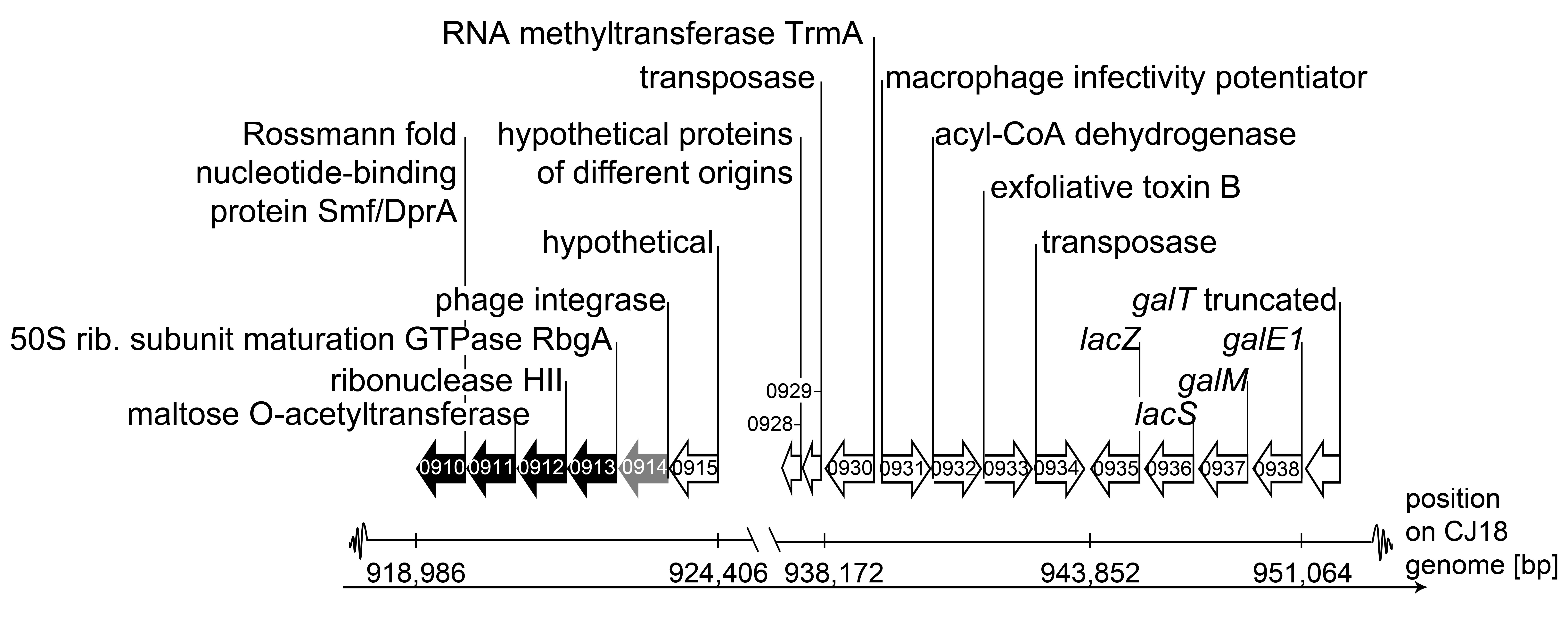

Supplement: Additional file 3 — Unique 13.2-kb gene locus with high DNA sequence identity to S. thermophilus in Sii genome CJ18. Description of data: The African Sii CJ18 harbors an approximately 13.2-kb insert of DNA with high sequence identity to S. thermophilus LMD-9 (white arrows) within a 25.6-kb insert (R6, Sinf_0915-Sinf_0939). The 18.4-kb gap between Sinf_0910 to Sinf_0928 was largely occupied with hypothetical proteins of unknown origin, few transporters and phage-related genes. Genes are not drawn to scale. Gene numbering corresponds to CDS region Sinf_0910-Sinf_0938. Black arrows indicate S. infantarius identity; grey other streptococci and white S. thermophilus identity. [file 1471-2164-14-200-S3.tiff]

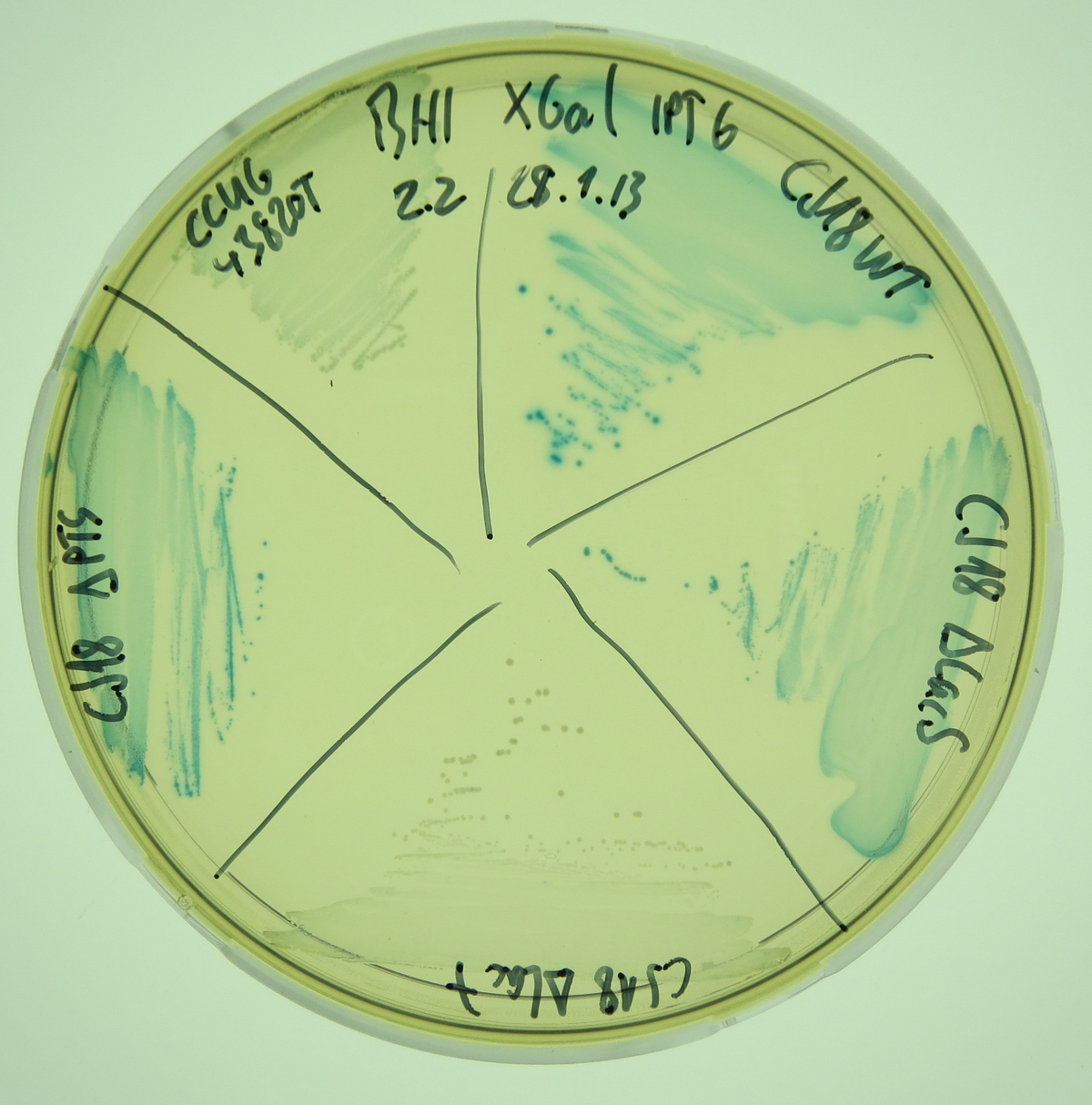

Supplement: Additional file 4 — Phenotypes of CJ18WT and mutant KO derivatives on BHI/X-Gal/IPTG agar media. Description of data: Confirmation of phenotypes of wild type, reference and KO strains on BHI/X-Gal/IPTG agar media yielding blue colonies for CJ18WT, CJ18ΔlacIIC, CJ18ΔlacS and white colonies for CJ18ΔlacZ and CCUG 43820T. [file 1471-2164-14-200-S4.jpeg]

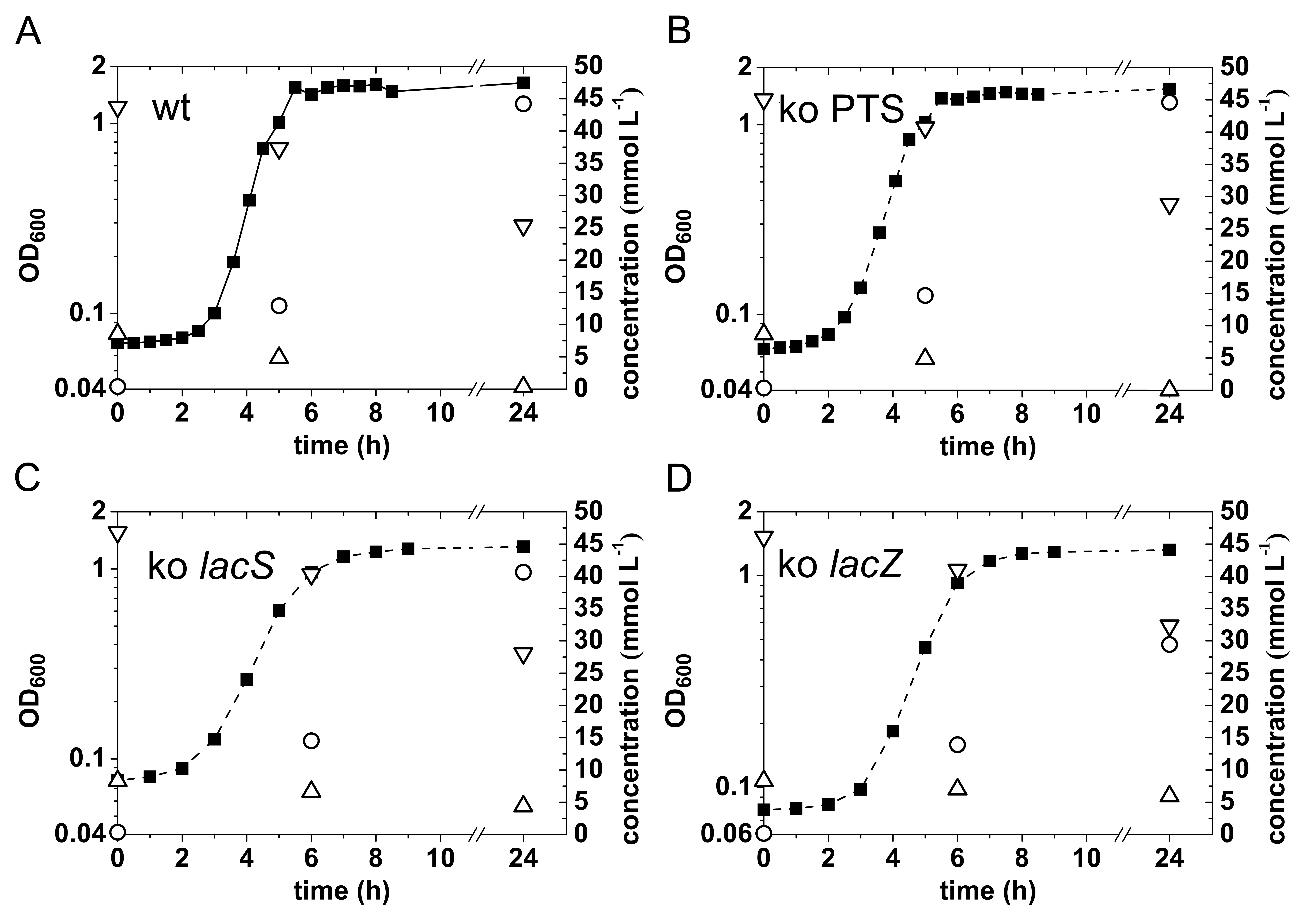

Supplement: Additional file 5 — Growth kinetics of wild type and knock-out strains of Sii CJ18 in glucose medium. Description of data: Growth kinetics of CJ18 wild type (A), CJ18ΔlacIIC (B), CJ18ΔlacS (C) and CJ18ΔlacZ (D) were compared in Elliker-based glucose medium for optical density (OD600 ■) and for metabolites glucose (∇),lactate (○) and galactose (ᐃ) in cell-free supernatant. Representative curves of two independent repetitions per strain are shown. [file 1471-2164-14-200-S5.tiff]
